# Evaluating the Impact of Sex-Specific Artificial Intelligence on Bone Metastasis Detection: VSBONE Bone Scan Index (BSI) Version 2.1 Versus Version 3.0

**DOI:** 10.7759/cureus.106562

**Published:** 2026-04-07

**Authors:** Aliya Toilybayeva, Reiko Ideguchi, Takashi Kudo

**Affiliations:** 1 Department of Radioisotope Medicine, Atomic Bomb Disease Institute, Nagasaki University Graduate School of Biomedical Sciences, Nagasaki, JPN; 2 Department of Radioisotope Medicine, Atomic Bomb Disease Institute, Nagasaki University, Nagasaki, JPN

**Keywords:** artificial intelligence, bone metastasis, bone scan index, bone scintigraphy, computer-aided diagnosis (cad), deep learning, nuclear medicine, sex-specific analysis

## Abstract

Objective(s): The present study compared the diagnostic accuracy of two different versions of an AI-assisted computer-aided diagnosis (CAD) software (VSBONE versions 2.1 and 3.0, Nihon Medi-Physics, Tokyo, Japan) for detecting bone metastases using bone scintigraphy images, with a focus on evaluating the impact of the new sex-specific analysis feature in version 3.0 on the overall performance and diagnostic accuracy in clinical decision-making.

Methods: This retrospective study analyzed 1,421 bone scintigraphy images from 1,119 patients at Nagasaki University Hospital. Patients were initially classified into metastatic and non-metastatic groups based on expert visual interpretation of planar images. Final classifications were made after single-photon emission computed tomography (SPECT)/CT and clinical review, resulting in metastatic, non-metastatic, and initially missed metastases groups. Bone Scan Index (BSI) and Hotspot number (HSn) were quantified using VSBONE BSI versions 2.1 and 3.0.

Results: Overall diagnostic performance improved with version 3.0 compared to version 2.1. In male patients, specificity increased (from 83.9% to 86.4%), with a slight decrease in sensitivity (from 82.0% to 79.7%), in the Youden index remained similar (from 0.660 to 0.662). In female patients, diagnostic accuracy improved, with notable increases in sensitivity (from 77.3% to 79.1%) and specificity (from 73.9% to 86.7%), and a marked increase in the Youden index (from 0.512 to 0.658).

Conclusion: The present study showed that version 3.0 improved diagnostic quality and accuracy, particularly in the sex-specific analysis (female patients). These findings support the integration of sex-specific AI in CAD software to enhance diagnostic accuracy, reduce interpretation errors, and promote more equitable patient care.

## Introduction

Bone scintigraphy is a fundamental method for detecting bone metastases. However, the visual interpretation of bone scintigraphy may be subjective and requires significant clinical expertise. To reduce this subjectivity and improve diagnostic accuracy, several methods and quantitative parameters have been introduced. One of the most well-known parameters is the Bone Scan Index (BSI), which represents the percentage of total skeletal mass involved by metastatic lesions and serves as a quantitative measure of skeletal metastatic burden and disease severity. BSI is automatically calculated based on the detection and quantification of abnormal hotspots on bone scintigraphy images [1,2]. However, the manual calculation of BSI may be time-consuming and require considerable expertise.

The development of computer-aided diagnosis (CAD) software (VSBONE BSI, Nihon Medi-Physics, Tokyo, Japan) has significantly contributed to enhancing the interpretation of medical images, including bone scintigraphy [3,4]. CAD software assists physicians in interpreting images and providing second opinions [4]. In recent years, artificial intelligence (AI), particularly deep learning, has been widely used in radiology for image processing. Machine learning involves training AI on large datasets, often under the guidance of experienced radiologists [4]. Computer programs for automatic medical image analysis, such as computer-aided diagnosis (CAD) software, are becoming increasingly important for bone scintigraphy [3].

In Japan, a CAD software program named VSBONE BSI has been developed to automatically detect and quantify bone metastases on bone scintigraphy images using deep learning methods [5].

The first version (ver.1) was introduced in 2019, followed by an improved version (ver.2.1) in 2022. Version 2.1 was trained using 1,034 cases consisting only of men with prostate cancer. The latest version (ver.3.0) was trained on an expanded dataset including both men with prostate cancer and women with breast cancer. The skeletal structure recognition model was trained using a combined dataset of 2,387 cases [6]. Therefore, the updated VSBONE version 3.0 covers data from a substantially larger dataset, including both male and female cases, significantly enhancing the capabilities and diagnostic accuracy of the analyses.

A recent study confirmed the diagnostic performance of VSBONE in a cohort of breast cancer patients, suggesting a significant improvement in the accuracy of version 3.0 compared to version 2.1 [7]. However, the previous study was limited to a single disease group (breast cancer) and did not examine the impact of sex differences or assess the applicability of the algorithm to broader clinical populations.

A key innovation of version 3.0 is the implementation of sex-specific modeling, which allows the algorithm to account for anatomical differences and differences in metastasis distribution between male and female patients [6]. The implementation of these sex-specific features may potentially enhance the diagnostic accuracy and clinical relevance of the VSBONE software in clinical decision-making.

The present study aimed to comprehensively compare the diagnostic performance of VSBONE versions 2.1 and 3.0 in differentiating metastatic from non-metastatic cases across a wide range of diseases, with a particular focus on the impact of sex-specific analysis in version 3.0.

## Materials and methods

Patients

The present study included 1,119 patients who underwent whole-body bone scintigraphy at Nagasaki University Hospital, Japan, between January 2015 and September 2020. Bone scintigraphy was performed using ^99m^Tc-hydroxymethylene diphosphonate (HMDP) or ^99m^Tc-methylene diphosphonate (MDP). Some patients underwent bone scintigraphy twice during the study period. Therefore, 1,421 bone scintigraphy images were analyzed in the present study. This study included patients with available bone scintigraphy images suitable for CAD analysis. Cases with incomplete imaging data or poor image quality were excluded. Bone scintigraphy images of patients with various diseases were investigated in this study. The majority of patients underwent bone scintigraphy for malignant disease and a survey for bone metastasis. However, other non-malignant diseases, such as Medication-related Osteonecrosis of the Jaw (MRONJ), were included.

Bone scintigraphy imaging

Patients underwent whole-body bone scans three to four hours after receiving an intravenous injection of 740 MBq of ^99m^Tc-HMDP (Nihon Medi-Physics, Japan) or ^99m^Tc-MDP (FUJIFILM Toyama Chemical/PDR Pharma, Japan). These scans were conducted using a dedicated single-photon emission computed tomography (SPECT)/CT scanner (Symbia T SPECT/CT scanner, Siemens Healthineers, Germany). Each patient underwent anterior and posterior whole-body planar imaging with a scan speed of 10 cm/min, using an energy window centered around the 140-keV energy peak and with a width of 15%. The resulting anterior and posterior images were subjected to analyses in this study. SPECT/CT images were also captured on the same scanner, employing a 128×128 matrix size and low-energy high-resolution parallel-hole collimators. These SPECT images, when fused with CT images, aided in identifying bone lesions corresponding to hot spots on whole-body bone scans, facilitating visual inspections for making the final diagnosis (positive or negative for bone metastasis). However, these SPECT/CT images were not used by CAD software for measurements and visual interpretation, which were compared with the CAD analysis.

Image analysis with visual interpretation

Patients were initially divided into non-metastatic and metastatic groups based on the visual interpretation of a skilled nuclear medicine physician with more than 30 years of experience without reviewing any information other than whole-body planar images. The use of a single reader was intended to maintain consistency in interpretation criteria across all cases. The interpreting physician was blinded to the CAD analysis results during this visual interpretation, as well as to the original medical reports and other clinical information. This visual interpretation was repeated twice, with more than a two-month interval, by the same physician. When two interpretations showed discordance, a third interpretation was performed and used as the final visual interpretation. After reviewing SPECT/CT images and medical reports, patients were divided into three groups: patients with metastatic disease (metastatic group), patients without metastatic disease (non-metastatic group), and patients with initially missed metastases confirmed on SPECT/CT (initially missed metastases group). The initially missed metastases group included patients who were initially classified as non-metastatic based on visual inspection of whole-body planar images but were ultimately diagnosed with bone metastases using SPECT, SPECT/CT fusion images, and other imaging modalities, such as conventional CT and MRI, and medical records. Because visual interpretation was performed by a single reader, inter-observer variability was not assessed in this study.

Image analysis using computer-aided diagnosis (CAD) software

Data analyses were performed using the deep learning neural network (trained) computer-aided diagnosis software VSBONE BSI (Nihon Medi-Physics, Tokyo, Japan) [5]. This software employs a deep learning network, specifically a butterfly-type fully convolutional network, to analyze anterior and posterior bone scintigraphy images using data obtained with^99m^Tc-HMDP [5,8]. However, in the present study, we also used images obtained with^ 99m^Tc-MDP. Both tracers are bisphosphonates that exhibit similar mechanisms of action and biodistribution in bone scintigraphy, enabling effective detection of bone lesions [9]. Despite some differences, their use is generally considered interchangeable in clinical practice in Japan.

VSBONE BSI is designed to quantitatively assess bone metastases in patients. Key features include Bone Scan Index (BSI) and Hotspot number (HSn). The software performs rapid calculations of these metrics by recognizing the anatomical structure of the skeleton and identifying areas of high radiopharmaceutical uptake [5]. It excludes physiological accumulation, such as the bladder, normalizes the display scale (concentration, size, and position), and sets an initial level of attention [6]. The CAD analysis was performed fully automatically by the software. No manual correction or operator verification of BSI delineation or hotspot detection was performed.

The most recent version of the software (version 3.0) was trained on a sex-specific (male/female) database, whereas the previous version (version 2.1) was trained on a male-only dataset. We herein use the terms version 3 and version 2 for these versions.

Ethical approval was granted by the Ethics Committee of Nagasaki University Hospital (Approval No.: 22031408), with a waiver of written informed consent in view of the retrospective nature of the study. All procedures conducted in the studies involving human participants were in accordance with the 1964 Helsinki Declaration and its later amendments or comparable ethical standards.

Statistical analysis

All statistical analyses were performed using EZR (Easy R; Saitama Medical Center, Jichi Medical University, Saitama, Japan) version 1.68, statistical software based on the R programming environment and R Commander. Because the same cases were analyzed by both software versions, paired Student's t-tests were used to evaluate differences between VSBONE version 2 and version 3. A p-value <0.05 was considered to be significant. 

Diagnostic performance was evaluated using sensitivity, specificity, and the Youden index. Sensitivity was calculated as true positives (TP)/(true positives + false negatives (FN)), and specificity as true negatives (TN)/(true negatives + false positives (FP)). The Youden index (J) was calculated as J = sensitivity + specificity - 1 and was used to determine the optimal threshold that maximized the sum of sensitivity and specificity. The optimal threshold was determined separately for each software version. Receiver operating characteristic (ROC) curves were generated by plotting the sensitivity and specificity of BSI. Sensitivity, specificity, and area under the ROC curve (AUC) were calculated with corresponding 95% confidence intervals.

## Results

Patient characteristics

Patient characteristics are shown in Table 1.

**Table 1:** Patient characteristics. HMDP: 99mTc-hydroxymethylene diphosphonate; MDP: 99mTc-methylene diphosphonate.

Characteristics	Value
Patients	1119
Duplicated cases	302
Total number оf bone scan images	1421
Non-metastatic group	1019 (71.7%)
Metastatic group	373 (26.2%)
Initially missed metastases group	29 (2.0%)
Age (years)	66.47 ± 21.02
Sex distribution	
Male	680 (60.8%)
Female	439 (39.2%)
Tracer information (per scan)	
^99m^Tc-HMDP	967 (68.1%)
^99m^Tc-MDP	454 (31.9%)
Main diseases	
Breast cancer	176 (15.7%)
Prostate cancer	396 (35.4%)
Renal cancer	150 (13.4%)
Lung cancer	48 (4.3%)
Urinary bladder cancer	39 (3.5%)
Hepatocellular carcinoma	37 (3.3%)
Medication-related Osteonecrosis of the Jaw (MRONJ)	6 (0.5%)
Other diseases	105 (9.4%)
Other cancer	162 (14.5%)

*Comparison of Bone Scan Index* *(BSI) and Hotspot Number (HSn)*

Table 2 shows a comparison between versions 2 and 3. BSI and HSn values are presented as mean ± standard deviation (SD), representing the average value and variability across patients in each group.

In the non-metastatic group, BSI of version 2 was significantly higher than that of version 3 (0.19 ± 0.52 vs. 0.12 ± 0.42, p < 0.0001); HSn of version 2 was significantly higher than that of version 3 (1.10 ± 2.51 vs. 0.86 ± 2.25, p < 0.0001). These results may be interpreted as an improvement in falsely detected positive findings, because BSI and HSn in the non-metastatic group were expected to be close to zero.

In the metastatic group, BSI did not significantly differ between versions 2 and 3 (2.63 ± 3.80 vs. 2.59 ± 3.89, p = 0.3432). However, HSn was significantly higher in version 3 compared to version 2 (15.01 ± 21.45 vs. 13.28 ± 17.19, p < 0.0001).

In the initially missed metastases group, BSI did not significantly differ between versions 2 and 3 (0.47 ± 1.01 vs. 0.32 ± 0.87, p = 0.0796). HSn also showed no significant difference between versions 2 and 3 (3.38 ± 6.26 vs. 2.65 ± 5.93, p = 0.0985).

**Table 2:** Comparison of BSI and HSn between version 2 and version 3 in non-metastatic, metastatic, and initially missed metastases groups. BSI: Bone Scan Index, HSn: hotspot number.

Group	Number of BS images	BSI%			HSn		
		Mean ± SD		p-value	Mean ± SD		p-value
		Version 2	Version 3		Version 2	Version 3	
Non-metastatic group	1019	0.19 ± 0.52	0.12 ± 0.42	<0.0001	1.10 ± 2.51	0.86 ± 2.25	<0.0001
Metastatic group	373	2.63 ± 3.80	2.59 ± 3.89	0.3432	13.28 ± 17.19	15.01 ± 21.45	<0.0001
Initially missed metastases group	29	0.47 ± 1.01	0.32 ± 0.87	0.0796	3.38 ± 6.26	2.65 ± 5.93	0.0985

Female Subgroup Analysis

Table 3 shows an analysis limited to females. In the non-metastatic group, BSI of version 2 was significantly higher than that of version 3 (0.25 ± 0.54 vs. 0.08 ± 0.25, p < 0.0001). HSn of version 2 was also significantly higher than that of version 3 (1.32 ± 2.08 vs. 0.67 ± 1.34, p < 0.0001).

In the metastatic group, BSI of version 2 was significantly higher than that of version 3 (1.73 ± 2.33 vs. 1.51 ± 2.36, p = 0.0390), while HSn did not significantly differ between versions 2 and 3 (9.28 ± 12.18 vs. 9.71 ± 15.84, p = 0.5828).

In the initially missed metastases group, there were no significant differences in BSI between versions 2 and 3 (0.54 ± 0.80 vs. 0.30 ± 0.55, p = 0.1870). HSn also did not significantly differ between versions 2 and 3 (3.42 ± 5.86 vs. 2.71 ± 4.90, p = 0.3200).

**Table 3:** Comparison of BSI and HSn between version 2 and version 3 in the non-metastatic, metastatic, and initially missed metastases groups for females. BSI: Bone Scan Index, HSn: hotspot number.

Group	Number of BS images	BSI%			HSn		
		Mean ± SD		p-value	Mean ± SD		p-value
		Version 2	Version 3		Version 2	Version 3	
Non-metastatic group	422	0.25 ± 0.54	0.08 ± 0.25	<0.0001	1.32 ± 2.08	0.67 ± 1.34	<0.0001
Metastatic group	67	1.73 ± 2.33	1.51 ± 2.36	0.0390	9.28 ± 12.18	9.71 ± 15.84	0.5828
Initially missed metastases group	14	0.54 ± 0.80	0.30 ± 0.55	0.1870	3.42 ± 5.86	2.71 ± 4.90	0.3200

Male Subgroup Analysis

Table 4 shows an analysis limited to males. In the non-metastatic group, BSI of version 2 was significantly higher than that of version 3 (0.16 ± 0.50 vs. 0.14 ± 0.50, p = 0.0092), whereas HSn did not significantly differ between versions 2 and 3 (0.96 ± 2.77 vs. 0.98 ± 2.70, p = 0.5527).

In the metastatic group, BSI did not significantly differ between versions 2 and 3 (2.83 ± 4.02 vs. 2.83 ± 4.11, p = 0.9242). However, HSn of version 3 was significantly higher than that of version 2 (16.17 ± 22.34 vs. 14.16 ± 18.00, p < 0.0001).

In the initially missed metastases group, there were no significant differences in BSI between versions 2 and 3 (0.41 ± 1.20 vs. 0.34 ± 1.10, p = 0.0508). HSn also did not significantly differ between versions 2 and 3 (3.33 ± 6.82 vs. 2.66 ± 6.92, p = 0.1879).

**Table 4:** Comparison of BSI and HSn between version 2 and version 3 in the non-metastatic, metastatic, and initially missed metastases groups for males. BSI: Bone Scan Index, HSn: hotspot number.

Group	Number of BS images	BSI%			HSn		
		Mean ± SD		p-value	Mean ± SD		p-value
		Version 2	Version 3		Version 2	Version 3	
Non-metastatic group	597	0.16 ± 0.50	0.14 ± 0.50	0.0092	0.96 ± 2.77	0.98 ± 2.70	0.5527
Metastatic group	306	2.83 ± 4.02	2.83 ± 4.11	0.9242	14.16 ± 18.00	16.17 ± 22.34	<0.0001
Initially missed metastases group	15	0.41 ± 1.20	0.34 ± 1.10	0.0508	3.33 ± 6.82	2.66 ± 6.92	0.1879

Comparison of Sensitivity and Specificity

In this comparison, shown in Table 5, only the non-metastatic and metastatic groups were included in the analysis of bone scintigraphy images. Sensitivity was higher in version 3 (85.8%) than in version 2 (81.1%), and specificity was also higher in version 3 (81.3%) than in version 2 (79.6%). The Youden index was higher in version 3 (0.671) than in version 2 (0.607), indicating a better overall balance between sensitivity and specificity in version 3. The AUC was also slightly higher in version 3 (0.895) than in version 2 (0.866).

**Table 5:** Comparison of sensitivity and specificity between version 2 and version 3 in non-metastatic and metastatic groups. CAD: computer-aided diagnosis.

CAD version	Mean±SD	Sensitivity (%)	Specificity (%)	Youden index	p-value
Version 2	0.85±2.28	81.1	79.6	0.607	<0.0001
Version 3	0.78±2.32	85.8	81.3	0.671	

Table 6 shows that for males, specificity was higher in version 3 (86.4%) than in version 2 (83.9%), whereas the sensitivity was slightly lower in version 3 (79.7%) than in version 2 (82.0%). The Youden index for males was slightly higher in version 3 (0.662) than in version 2 (0.660), reflecting a slight improvement in the balance between sensitivity and specificity. The AUC was slightly higher in version 3 (0.897), but very close to that in version 2 (0.886).

For females, version 3 showed improvements across all key metrics. Sensitivity was higher in version 3 (79.1%) than in version 2 (77.3%), and specificity was significantly higher in version 3 (86.7%) than in version 2 (73.9%). The Youden index for females was markedly higher in version 3 (0.658) than in version 2 (0.512), reflecting a significant improvement in overall diagnostic performance for females. The AUC was higher in version 3 (0.871) than in version 2 (0.823).

**Table 6:** Comparison of sensitivity and specificity between version 2 and version 3 in non-metastatic and metastatic groups by sex. CAD: computer-aided diagnosis.

CAD version	Mean ± SD	Sensitivity (%)	Specificity (%)	Youden index
Male				
Version 2	1.06 ± 2.69	82.0	83.9	0.660
Version 3	1.05 ± 2.74	79.7	86.4	0.662
Female				
Version 2	0.45 ± 1.11	77.3	73.9	0.512
Version 3	0.28 ± 1.02	79.1	86.7	0.658

**Figure 1:**
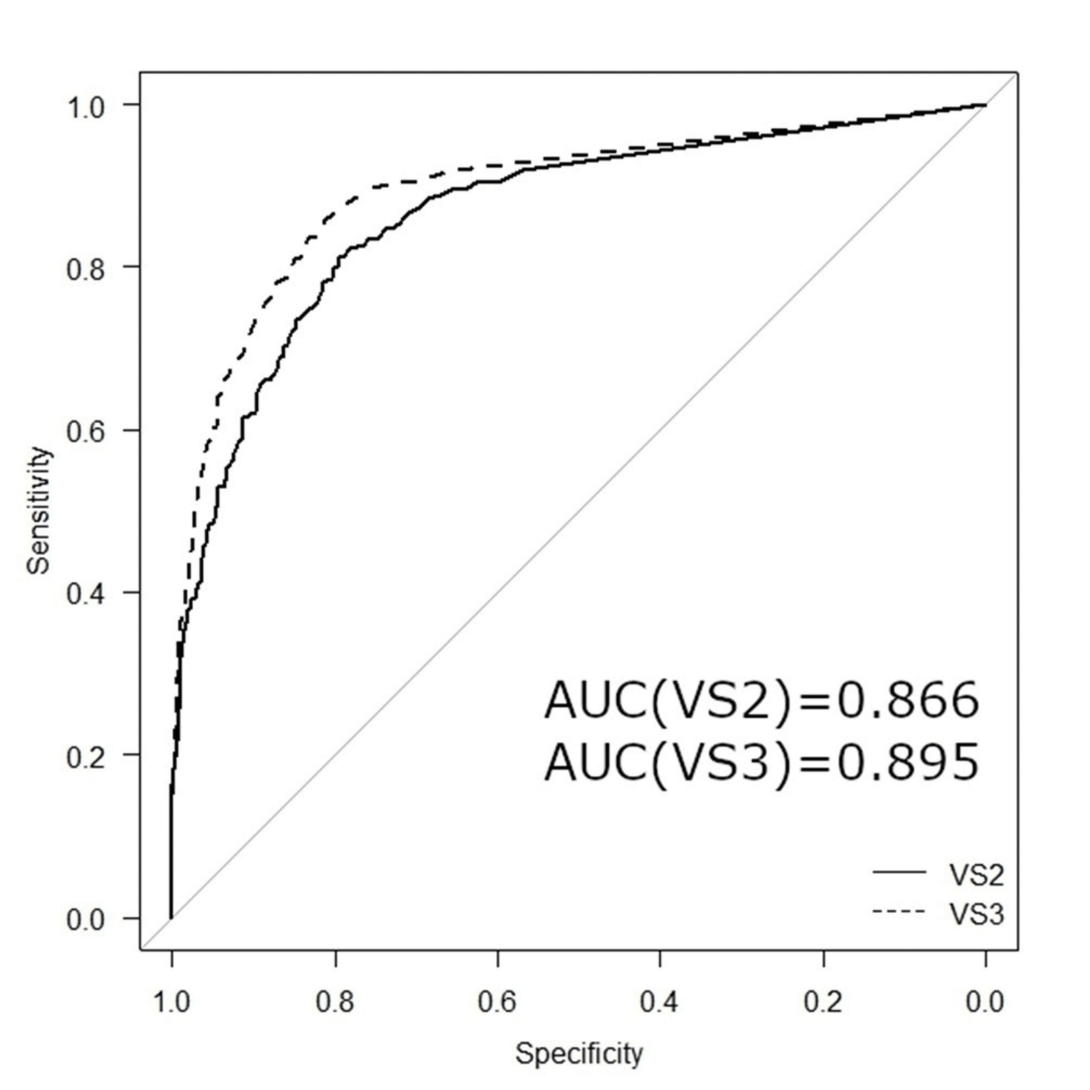
Comparison of two ROC curves of VSBONE between version 2 (VS2) and 3 (VS3). ROC: receiver operating characteristics, AUC: area under the curve.

Figure 1 shows a comparison of ROC curves for BSI between version 2 and version 3 without sex differentiation. The AUC for version 2 was 0.866, while that for version 3 was slightly higher at 0.895.

**Figure 2:**
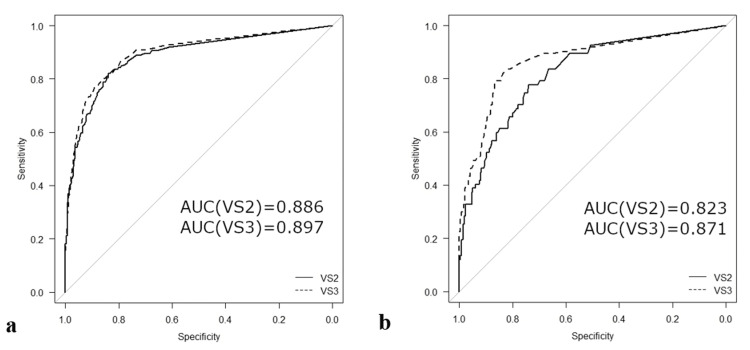
Comparison of two ROC curves of VSBONE between version 2 (VS2) and version 3 (VS3) according to sex: (a) male and (b) female. ROC: receiver operating characteristics, AUC: area under the curve.

Figure 2(a) (males) shows that the AUC for version 2 was 0.886, while that for version 3 was slightly higher at 0.897. In Figure 2(b) (females), the AUC for version 2 was 0.823, and that for version 3 was higher at 0.871.

## Discussion

In the present study, we compared the effectiveness of two versions of the VSBONE CAD software, specifically versions 2 and 3, for detecting metastatic lesions on bone scintigraphy images. The results showed several key findings regarding the diagnostic accuracy and overall efficiency of these versions. The comparison between VSBONE 2 and 3 shows that version 3 offers improvements in diagnostic parameters. In non-metastatic cases, BSI and HSn were significantly lower in version 3 than in version 2, indicating fewer false positives. In metastatic cases, while BSI remained unchanged, version 3 identified more hot spots, which may reflect improved detection of metastatic lesions, although differences in segmentation between software versions cannot be excluded. The number of initially missed metastases in the group was slightly lower with version 3.

Overall, the present study showed that version 3 had higher sensitivity (from 81.1% to 85.8%) and specificity (from 79.6% to 81.3%), indicating improved performance in distinguishing between metastatic and non-metastatic cases. 

These results suggest that version 3 of VSBONE may be more reliable for clinical use, potentially supporting more accurate diagnoses and treatment decisions through improved interpretation of bone scintigraphy findings. The implementation of more advanced and sophisticated machine learning algorithms allowed the program to better identify and quantify metastatic lesions. These algorithms became more effective due to the expansion of the training dataset, particularly by including data that accounts for sex differences.

Sex-specific analyses indicate that version 3 improved diagnostic efficiency for women, with sensitivity increasing (from 77.3% to 79.1%), and specificity showing a marked improvement (from 73.9% to 86.7%). The Youden index also markedly increased (from 0.512 to 0.658). This improvement in the Youden index suggests that the updated tool provides a better balance between sensitivity and specificity, offering the more accurate detection of metastatic cases while also improving the correct identification of non-metastatic conditions. These findings suggest that the sex-specific training implemented in version 3 particularly improved diagnostic accuracy for women, primarily by reducing false positive classifications, while overall diagnostic performance in men remained largely comparable between versions, as the slight decrease in sensitivity was offset by an increase in specificity. These results are consistent with previous reports that the inclusion of female skeletal structures into the CAD model training significantly reduced the false positive rate in diagnostic evaluation in the pelvic region and improved the overall diagnostic performance of VSBONE 3 (sensitivity 90.0%; specificity 79.5%; accuracy 80.5%) in breast cancer patients [7].

The performance improvements in version 3 are likely due to both algorithmic refinements and the expansion of the training database. By including data from both male and female patients and accounting for anatomical and metabolic differences between the sexes, the updated version of VSBONE was able to better interpret tracer accumulation patterns characteristic of female skeletal anatomy. These results align with prior reports showing that unbalanced training datasets can lead to systematic bias. Larrazabal et al. reported that datasets biased towards male patients reduced the effectiveness of CAD software for diagnosing women, emphasizing the importance of ensuring sex diversity in the medical data used to train these softwares [10]. Additionally, Seyed-Kalantari et al. reported that AI models trained on unbalanced chest X-ray datasets tend to underdiagnose female patients and patients from disadvantaged socioeconomic groups [11]. Therefore, CAD software trained without adequate sex representation is likely to be less accurate for women if they are underrepresented in the training data. According to research, 26-50% of patients with breast cancer are at a high risk of developing bone metastases [12]. Improving diagnostic accuracy in female patients may therefore help reduce false positive findings and unnecessary follow-up examinations in clinical practice. This finding highlights the importance of the sex-specific training of AI models to improve diagnostic accuracy.

**Figure 3:**
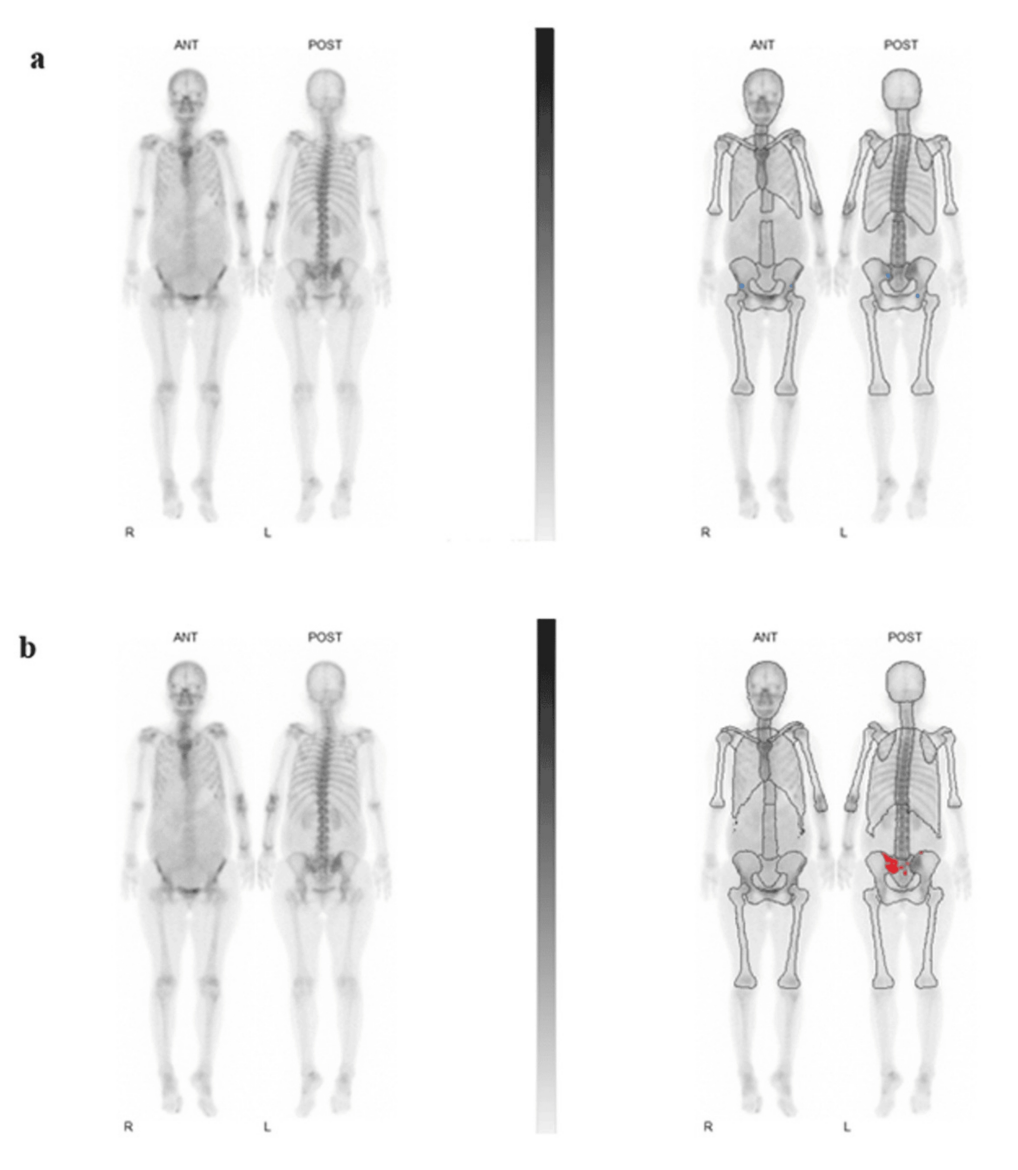
Bone scintigraphy imaging using a CAD software for a 61-year-old woman with hepatocellular carcinoma, initially classified as a false negative. The red-marked area indicates the area that VSBONE considered to be metastasis. VSBONE 2 (a) showed no abnormalities (BSI 0.00%, HSn 0), while VSBONE 3 (b) detected metastasis in the sacrum and pelvis (BSI 0.98%, HSn 5). Image is a direct output of the VSBONE BSI clinical computer-aided diagnosis (CAD) software (Nihon Medi-Physics Co., Ltd., Tokyo, Japan), displaying actual bone scintigraphy images of a 61-year-old patient with hepatocellular carcinoma. BSI: Bone Scan Index; HSn: hotspot number.

The present study also analyzed a small group of patients with initially missed metastases confirmed on SPECT/CT, for which the visual interpretation of a planar image failed to detect metastases, even by an experienced interpreter; however, they were identified using VSBONE software, as shown in Figure 3. These results suggest that while CAD software significantly reduces the burden of manual calculations, it may also serve as a valuable tool for minimizing image misinterpretation, effectively acting as a secondary check for radiologists. False-negative interpretations may result in critical failure in the daily management of patients with malignancies. In some malignancies, such as stage I-II breast cancer, Schnipper et al. reported that the early detection of metastasis did not necessarily impact the prognosis of patients [13]. However, Koizumi et al. showed that missing existing bone metastases during a visual inspection naturally resulted in delayed treatment and reduced quality of life [14]. In this context, it is very important to have a tool that avoids false-negative findings. Urano et al. indicated that CAD software needs further improvement to reduce false-negative results. However, VSBONE increased sensitivity from 40.8% with planar imaging alone to 50.2%; it still missed many metastatic lesions [15]. Improved detection is essential to reduce patient risk from undiagnosed metastases.

In the present study, we used the radiopharmaceuticals ^99m^Tc-MDP and ^99m^Tc-HMDP, which are commonly employed in bone scintigraphy to detect metastases in prostate cancer. Higashiyama et al. reported that the BSI and HSn parameters obtained using ^99m^Tc-HMDP with VSBONE can be used as clinically useful quantitative indicators similar to those obtained from ^99m^Tc-MDP images analyzed with BONENAVI (FUJIFILM RI Pharma Co.) [16]. These findings indicate that BSI values generated with either tracer are consistent and can be used interchangeably in clinical practice, as both ^99m^Tc-MDP and ^99m^Tc-HMDP share the same mechanism of accumulation in bone tissue and have demonstrated comparable BSI values for assessing metastatic disease.

Limitations

There are several limitations that need to be addressed. VSBONE version 3 achieved better sensitivity and specificity; however, there is the potential for further improvements by reducing errors and increasing diagnostic accuracy. In addition, both ^99m^Tc-MDP and ^99m^Tc-HMDP bone scans were included in this study, whereas VSBONE BSI was initially trained on ^99m^Tc-HMDP scans. Although both tracers share similar mechanisms of bone uptake, this difference may cause slight variability, which requires further research. In addition, the effectiveness of CAD software, such as VSBONE version 3, depends heavily on the ability of radiologists to incorporate these results into clinical practice.

## Conclusions

The present study provides significant information for the development and application of CAD software in medical diagnostics. VSBONE version 3 has improved diagnostic capabilities, particularly in sex-specific analyses and overall diagnostic accuracy. These results emphasize the importance of continued research on sex-specific CAD software training and the further integration of VSBONE into clinical practice to improve diagnostic quality while also promoting more reliable and equitable patient care and reducing interpretation errors.
